# There and back again: 50 years of wandering through *terra incognita fusorum*

**DOI:** 10.1113/EP090760

**Published:** 2023-01-11

**Authors:** Robert W. Banks

**Affiliations:** ^1^ Department of Biosciences University of Durham Durham UK; ^2^ Biophysical Sciences Institute University of Durham Durham UK

**Keywords:** equatorial nucleus, intrafusal fibre, muscle spindle, primary sensory ending

## Abstract

This paper is in two parts: ‘There’, which is a review of some of the major advances in the study of spindle structure and function during the past 50 years, serving as an introduction to the symposium entitled ‘Mechanotransduction, Muscle Spindles and Proprioception’ held in Munich in July 2022; and ‘And Back Again’, presenting new quantitative morphological results on the equatorial nuclei of intrafusal muscle fibres and of the primary sensory ending in relationship to passive stretch of the spindle.

## Abstract (highlights)

- **What is the topic of this paper?**
  This paper concerns the relationship between structure and function of the mammalian muscle spindle, its intrafusal muscle fibres and their sensory and motor innervation.
- **What advances does it highlight?**
  The paper summarizes the development of knowledge of the number of types of intrafusal muscle fibres and the pattern and functional significance of their fusimotor innervation. New observations are presented on the quantitative functional morphology of equatorial nuclei and primary sensory endings.

## 1 THERE … REFLECTIONS ON WHAT WE KNEW AND DID NOT KNOW 50 YEARS AGO, AND SOME OF WHAT WE HAVE LEARNT SINCE

Fifty years ago, I was writing my PhD thesis, which was submitted the following year to the Faculty of Medicine in the University of Sheffield. The previous 3 years of my graduate studies had been carried out in the Department of Human Biology and Anatomy, where I had first been introduced to the mammalian muscle spindle and where I had researched its structure and innervation, using enzyme histochemistry, serial sectioning of epoxy‐resin‐embedded tissue, transmission electron microscopy and silver‐impregnated whole spindles. The background to my work was a model of the mammalian spindle that had arisen in the early 1960s from two particularly influential papers, the first by Ian Boyd (Boyd, 1962) and the second by Peter Matthews (Matthews, 1962). By that time, the broad structural features of the spindle and of its innervation by both sensory and specific fusimotor neurons were well known, as reviewed by Sibyl Cooper (Cooper, 1960), and it was becoming increasingly apparent that the highly specialized muscle fibres that constitute the intrafusal bundle were not a single, homogeneous type. In particular, the accumulation of nuclei under the terminals of the primary sensory ending could adopt either a bag‐ or chain‐like arrangement, giving rise to the descriptive terms of nuclear‐bag fibre and nuclear‐chain fibre. Based on a large amount of evidence derived from several hindlimb muscles of the cat, Boyd (1962) concluded that ‘…from this work it is quite certain that mammalian muscle spindles contain two distinct types of intrafusal muscle fibre’. But he went beyond that ‘certain’ conclusion, further claiming that the two types of intrafusal fibre were separately innervated by fusimotor axons and elevating them to ‘the nuclear bag muscle fibre system’ and ‘the nuclear chain muscle fibre system’ (Boyd, 1962).

Peter Matthews, meanwhile, differentiated two functional kinds of fusimotor axon according to their actions on the response of the primary ending while it was being stretched (the phasic or ‘dynamic’ response; Matthews, 1962). In general, stimulation of fusimotor axons enhances the output of the primary ending at constant length (the tonic or ‘static’ response), but only some of the axons selectively enhance the dynamic response. In his paper, Peter concluded that: ‘There exist fusimotor fibres of two functionally distinct kinds, which for the purposes of the present description, and for the want of better terms, will be called static and dynamic fusimotor fibres’ (Matthews, 1962). The congruity between Ian Boyd's histological and his own physiological observations did not, of course, escape Peter, although in his review published 2 years later (Matthews, 1964) he was careful to point out that this did not constitute definitive proof of Ian's scheme of two separate systems, which was consistently challenged by David Barker (e.g., Barker & Cope, 1962). Nevertheless, a two‐element model in which nuclear‐bag fibres were thought to be innervated by dynamic fusimotor axons and nuclear‐chain fibres by static fusimotor axons was very soon adopted widely. In his review, moreover, Peter summarized Ian's conclusions in what was to become a highly influential schematic diagram (Figure 1 of Matthews, 1964); it has frequently been referred to in the secondary literature, where the notion of only two types of intrafusal fibre, nuclear bags and nuclear chains, lives on despite being as much as 40 years out of date (e.g. Purves et al., 2018).

But in the face of new evidence from electron microscopy and, more especially, from enzyme histochemistry, reviewed in the introduction to my thesis (Banks, 1973), the inadequacy of the dual morphological model was becoming increasingly apparent even as it was becoming widely accepted. On the basis of my own work, in the final discussion, I was able to conclude: ‘The results of the present study indicate that there are three types of intrafusal fibre in spindles of both guinea pig and rabbit’. Nevertheless, the number of types of intrafusal fibre and the particular properties of those types remained uncertain owing to the difficulties of correlating the results of the various techniques used. We (Banks et al., 1977) then published a study that combined light microscopical histochemistry with transmission electron microscopy on serially sectioned muscles and so provided a direct correlation between these important techniques. We found that in rat, rabbit and cat spindles the same three types of fibre were identifiable, despite interspecific differences in their numbers and sizes. These types, now generally known as bag_1_, bag_2_ and chain, continue to be recognized as a common feature of mammalian muscle spindles, but much important work on their properties is still being carried out, now considerably extended by the introduction of immunohistochemical methods (Thornell et al., 2015).

In our 1977 paper, we concluded that ‘The implications of the new classification are far reaching and earlier results may need to be re‐interpreted, particularly with regard to the innervation of the muscle spindle’. Throughout the 1970s, the motor innervation, in particular, proved to be a complex and often contentious problem of muscle spindle organization, but even when it was widely accepted that there were only two kinds of intrafusal fibre, Ian Boyd's notion that each received a separate motor supply was disproved by a collaborative study between David Barker's and Yves Laporte's laboratories showing that static γ axons usually innervated both nuclear‐bag and nuclear‐chain fibres often, although not necessarily, in the same spindle (Barker et al., 1973).

The typical highly branched nature of these static γ axons within a single spindle polar region could perhaps be seen to conform to Boyd's ‘γ2 network’ and to Barker's ‘trail ending’, but what of the dynamic γ axons? To address this question, David Barker devized another collaborative experiment, this time with Paul Bessou, in which the site of focal contraction seen in a living spindle on stimulation of a dynamic axon would be examined histologically to determine the nature of the nerve terminal presumed to occur there. In most cases, however, the anticipated ending was not found at the contraction site of the identified intrafusal muscle fibre, and in order to locate motor endings wherever they might occur we resorted to serial sectioning, at 1 μm thickness, of the whole spindles, which had been fixed and embedded in epoxy‐resin for electron microscopy (Banks et al., 1978). Not only did this approach reveal the activated dynamic axon and its ending or endings, but now the entire innervation of the whole spindle could be reconstructed (Banks, 1981; Banks et al, 1981). In the spindles whose innervation was reconstructed in this way, the fibres activated by the dynamic γ axons were found to be bag_1_ fibres; their motor innervation was almost invariably exclusive and separate from that of the bag_2_ and chain fibres, which were often supplied by the same, presumably static, axons.

The distribution of static γ axons to both bag_2_ and chain fibres was a notable exception to the notion of homogeneous motor units in mammalian skeletal muscle, although the frequent occurrence of skeletofusimotor axons supplying both intrafusal and extrafusal fibres, the so‐called β axons, was also becoming well known (e.g., Emonet‐Dénand & Laporte, 1975). But the distribution of static γ axons is entirely intrafusal, so why should they regularly supply two very different types of fibre? Perhaps it was even possible that the single category of static γ axon was too broad and might be concealing subdivisions of some kind. This was the view of the Glasgow school, led by Ian Boyd, who argued that there were two or even three (sub)types of static γ (e.g., Boyd, 1986). My own work contradicted this. Using the tenuissimus preparation to record as many combinations of static γ axons and Ia afferents in a single muscle as possible, I concluded that there was only one type of static γ, but the distributions of the axons were correlated, in part, with their conduction velocities; more rapidly conducting axons tended to be distributed to more spindles than slower ones and were less likely to supply chain fibres alone in any particular spindle (Banks, 1991). My conclusion was extended and borne out by Emonet‐Dénand et al. (1997), who showed that coactivation of bag_2_ and chain fibres allows the primary ending to signal changes of length over a large range of stretch velocities regardless of the average muscle length, and Emonet‐Dénand et al. (1998), who showed that static γ axons supplying bag_2_ fibres alone or together with chain fibres tended to have faster conduction velocities than those supplying chain fibres alone.

Details of the responses of primary endings to stimulation of a single static γ axon depended on whether bag_2_ or chain fibres or both were activated by the axon (Banks, 1991; Boyd et al., 1985), thus indicating the importance of the differing mechanical properties of the two types of intrafusal fibre. Many secondary endings, especially those adjacent to the primary (S1 secondaries), also have well‐developed terminals on the bag_2_ fibre in addition to their extensive chain‐fibre terminals (Banks et al., 1982), and their responses, too, depend on whether one or both types of fibre are activated (Boyd et al., 1985).

But there is a further complication with the secondary ending, which is probably of much greater importance from a technical, experimental point of view than a functional one. It is that a secondary ending in a spindle whose primary ending might be activated readily by a static γ axon might be located in the same pole as the motor endings of the axon or in the opposite pole. If the secondary and motor endings are in opposite poles, the response of the secondary can be affected only weakly or not at all by the static γ activation (Boyd et al., 1985; Celichowski et al., 1994).

**FIGURE 1:**
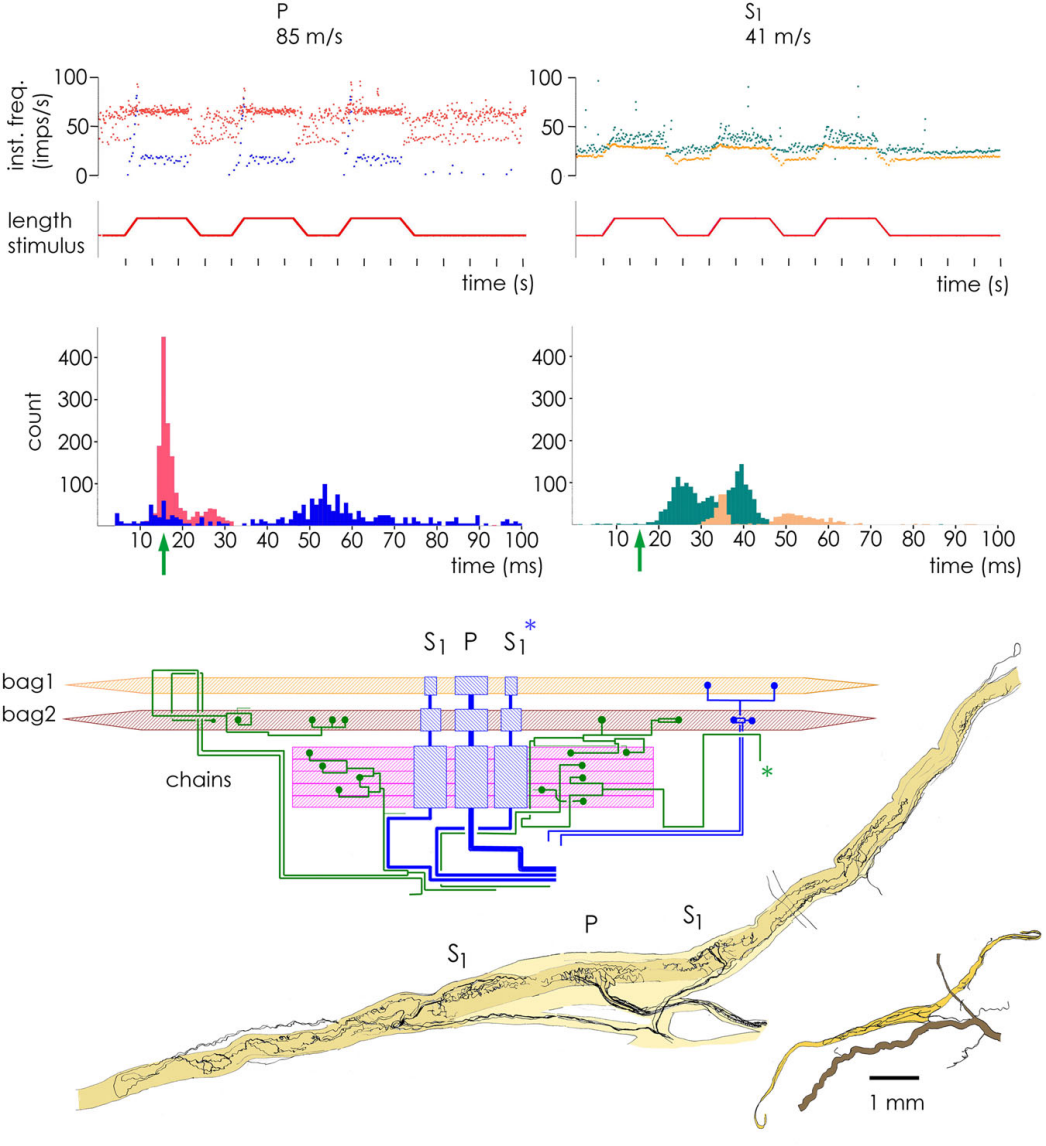
An example of simultaneous recording of a primary (P) and an S_1_ secondary in the same spindle (unpublished experiment, cat tenuissimus C933, preparation as in the study by Banks, 1991). Top row: instantaneous frequency plots of responses to three successive ramp, hold and release stretches with (red, green) and without (blue, yellow) stimulation of a single static γ (conduction velocity 41 m/s) at constant rate of ∼65 impulses/s. Middle row: corresponding interval histograms. The vertical arrow indicates the γ axon interstimulus interval. Insecure driving of P indicates bag_2_–chain co‐activation. Bottom: schematic diagram of the innervation of the spindle, together with camera lucida tracings, showing the innervated portion of the spindle and the entire spindle with the intramuscular nerve. The green asterisk indicates the likely activated γ axon, identified as the only fusimotor axon with extensive bag_2_ and chain distribution; the blue asterisk indicates the likely recorded S_1_ secondary, identified as being activated in the same pole as the γ distribution.

So far, so straightforward; but one other feature of the response of the secondary when a static γ axon activates chain fibres still eludes an obvious explanation, at least to my mind. Stimulation of such an axon between ∼50 and 100 impulses/s often produces ‘driving’ of the primary ending at the same frequency as the stimulation, which is attributable to the unfused tetanic contraction of the chain fibres, yet a secondary ending in the same pole as the activated chain fibres is only rarely driven in this way, although the regularly fluctuating tension of the chain fibres must be transmitted through the secondary ending to the primary (Figure 1). This is perhaps attributable, in part, to the relatively low sensitivity of the secondary ending to a rapidly changing mechanical stimulus, in comparison to the primary response and as is well known (Cooper, 1961). The differing structures of the primary and secondary endings, and especially of their terminals (Banks et al., 1982), in addition to differences in their ion channels, especially, perhaps, voltage‐ and Ca^2+^‐dependent K^+^ channels (Oliver et al., 2021; Wu et al., 2021), might all be contributing factors, but the inherent mechanical asymmetry of the secondary ending in relationship to the primary ending might also contribute, because it is known that many secondaries can be driven by a vibratory stimulus applied to the belly of a muscle, but not when the same stimulus is applied to the tendon (Bianconi & Van Der Meulen, 1963).

The various ion channels in spindle sensory terminals bring us into the complex process of mechanosensory transduction, which has been my main concern for the last 20‐odd years in collaboration with Guy Bewick (Bewick & Banks, 2015, 2021a, 2021b). We have been characterizing an autocrine glutamatergic system that appears to provide local control of the excitability of the sensory terminals, perhaps by insertion and sequestration of stretch‐sensitive and other ion channels into and from the terminal membrane by the recycling of synaptic‐like vesicles (Bewick & Banks, 2015, 2021b). We regard the identity of the ion channels themselves as ‘still an open question’ (Bewick & Banks, 2021a).

## 2 …AND BACK AGAIN: ON RECONSTRUCTING THE PRIMARY ENDING, WITH SOME RECENT OBSERVATIONS ON THE ENDING AND THE EQUATORIAL NUCLEI

### 2.1 Introduction

Tucked inside the back cover of my PhD thesis is a set of tracings on photographic film of the outlines of the intrafusal muscle fibres of a rabbit tenuissimus spindle, showing the location and extent of the equatorial nuclei (Banks, 1973). The spindle had been sectioned longitudinally for electron microscopy, and the tracings were made from intervening semithin (1‐μm‐thick) sections stained with Toluidine Blue for light microscopy. They were then stacked together to make a simple reconstruction of the equatorial and juxta‐equatorial regions. Since that first tentative step, I have made extensive use of reconstructions from serial semithin sections in my attempts to understand the relationship of structure and function in the muscle spindle.

To follow the distribution of fusimotor axons and to locate their terminals had necessitated the use of serial transverse sections, as mentioned above (Banks et al., 1978). For those purposes it was sufficient to generate schematic reconstructions, in which only features related to the long axis of the spindle needed to be shown to scale, but our interest in details of the form of the terminals required a more fully developed, graphical isometric technique (Banks, 1981). It was clear that the same isometric technique could be used to examine sensory endings in unprecedented detail, in particular revealing characteristic features of primary endings associated with each of the three types of intrafusal fibres. The serial sections themselves facilitated quantitative data on the contact area between sensory terminals and intrafusal muscle fibres and on numbers of underlying nuclei in the nuclear bags, myotubes and nuclear chains, for example (Banks et al., 1982).

**FIGURE 2:**
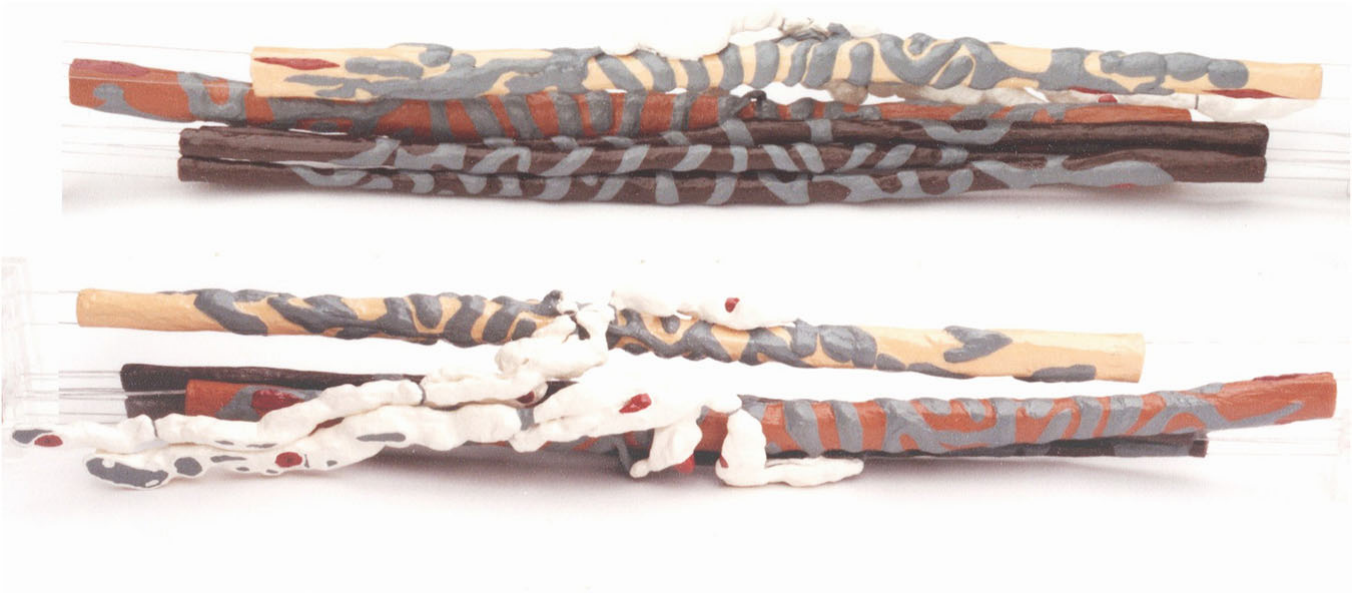
Two views of a fully three‐dimensional reconstruction of a cat tenuissimus P ending, originally published in the study by Banks (1986). Colours are as follows: grey, sensory terminals and other axonal structures; white, myelin; red, Schwann cell nuclei; yellow, bag_1_; light brown, bag_2_; dark brown, chains (3).

But the use of graphical isometric reconstruction and transverse sections raised additional questions about the form of the primary‐ending terminals and whether there was any detectable deformation of the terminals under different amounts of maintained (static) stretch of the spindles. To address these, I used serial longitudinal sections and a truly three‐dimensional solid reconstruction (Banks, 1986; Figure 2). Once again, the spindles had formed part of our earlier study on the distribution of motor axons (Banks et al., 1978) and had not been fixed systematically at different lengths. Nevertheless, the terminals did seem to be measurably deformed under different amounts of static stretch, as assessed by sarcomere lengths in the adjacent juxta‐equatorial regions, and they appeared to be compressed between the underlying surface of the muscle fibre and the overlying basal lamina. In recent years, advances in digital photography and computing have enabled virtual, interactive reconstruction, with inherent quantification, and I revisited one of the primary endings from the original graphical reconstruction of Banks et al. (1982) in my personal perspective review of the innervation of the muscle spindle (Banks, 2015).

The following results present new data from left and right pairs of tenuissimus muscles of the cat hindlimb, fixed in situ by perfusion with the left limb fully extended at the hip and knee, and the right limb flexed to varying degrees at each joint. Attention is focused on the equatorial nuclei and primary‐ending terminals in relationship to intrafusal muscle fibre type and to sarcomere length as a proxy for static stretch.

### 2.2 Methods

Left and right tenuissimus muscles were prepared in 1988 from three adult cats that formed part of a programme of work on sensory reinnervation being carried out in David Barker's laboratory under the Animals (Scientific Procedures) Act, 1986 (Adal & Banks, 1990; Banks & Barker, 1989). Each animal was given a lethal dose of sodium pentobarbitone (Sagatal, i.p.) and, immediately after cessation of respiration, was perfused transcardially with heparinized mammalian Ringer solution, followed by a standard Karnovsky fixative, with the right and left hip and knee joints flexed or extended as in Table 1. After removal, each tenuissimus muscle was cut approximately into proximal, middle and distal thirds and post‐fixed with 1% OsO_4_ before dehydration and embedding in epoxy‐resin. The blocks were then stored until examples of spindles from each portion of the six muscles were serially sectioned longitudinally at 1 μm thickness and stained with Toluidine Blue for light microscopy. The sectioning was carried out by my PhD student Abdulaziz Alamri, as described in his thesis (Alamri, 2004), and he made measurements of the profiles of terminals, as in my earlier paper (Banks, 1986), but he did not attempt any reconstructions.

**TABLE 1:** Summary of data for three pairs of cat tenuissimus muscles and of the mean sarcomere lengths and numbers of equatorial nuclei in the intrafusal fibres of the seven muscle spindles whose equatorial regions were reconstructed from serial, longitudinal sections of the muscles.

	Right limb (flexed)			Left limb (extended)			
Experiment	Hip angle (°)	Knee angle (°)	Length (mm)	Hip angle (°)	Knee angle (°)	Length (mm)	Flexed/extended (%)
866	55	45	102	130	155	150	68
872	95	87	125	130	155	152	82
869	75	85	141	140	145	148	95

Mean sarcomere length (μm)							
	Right limb (flexed)			Left limb (extended)			
	Bag_1_	Bag_2_	Chain	Bag_1_	Bag_2_	Chain	
866							
Proximal (A)	–	–	–	2.14	2.31	1.78	
Middle (B)	2.03	1.7 1.75	1.76	–	–	–	
Distal (C)	3.08	2.5	2.29	2.6	2.67 2.61	2.27	
872							
Proximal (A)	2.4	2.5	1.96	–	–	–	
Middle (B)	2.49	2.47	1.84	–	–	–	
869							
Proximal (A)	2.14	2.5	1.95	–	–	–	

Number of equatorial nuclei							
	Right limb (flexed)			Left limb (extended)			
	Bag_1_	Bag_2_	Chain	Bag_1_	Bag_2_	Chain	Total
866							
Proximal (A)				86	78	102 (4 fibres)	266
Middle (B)	94	86 87	218 (6 fibres)	–	–	–	485
Distal (C)	69	78	103 (6 fibres)	45	64 53	124 (6 fibres)	250 (right) 286 (left)
872							
Proximal (A)	77	73	122 (4 fibres)	–	–	–	272
Middle (B)	84	101	159 (5 fibres)	–	–	–	344
869							
Proximal (A)	86	99	137 (5 fibres)	–	–	–	322

The sections remained in good condition, and in 2019 I began a systematic digital photographic recording of all the complete equatorial regions using a Leica DM2500 equipped with a ×63 oil immersion objective. Reconstructions were made using freehand tracing in the program RECONSTRUCT (Fiala, 2005).

### 2.3 Results

**FIGURE 3:**
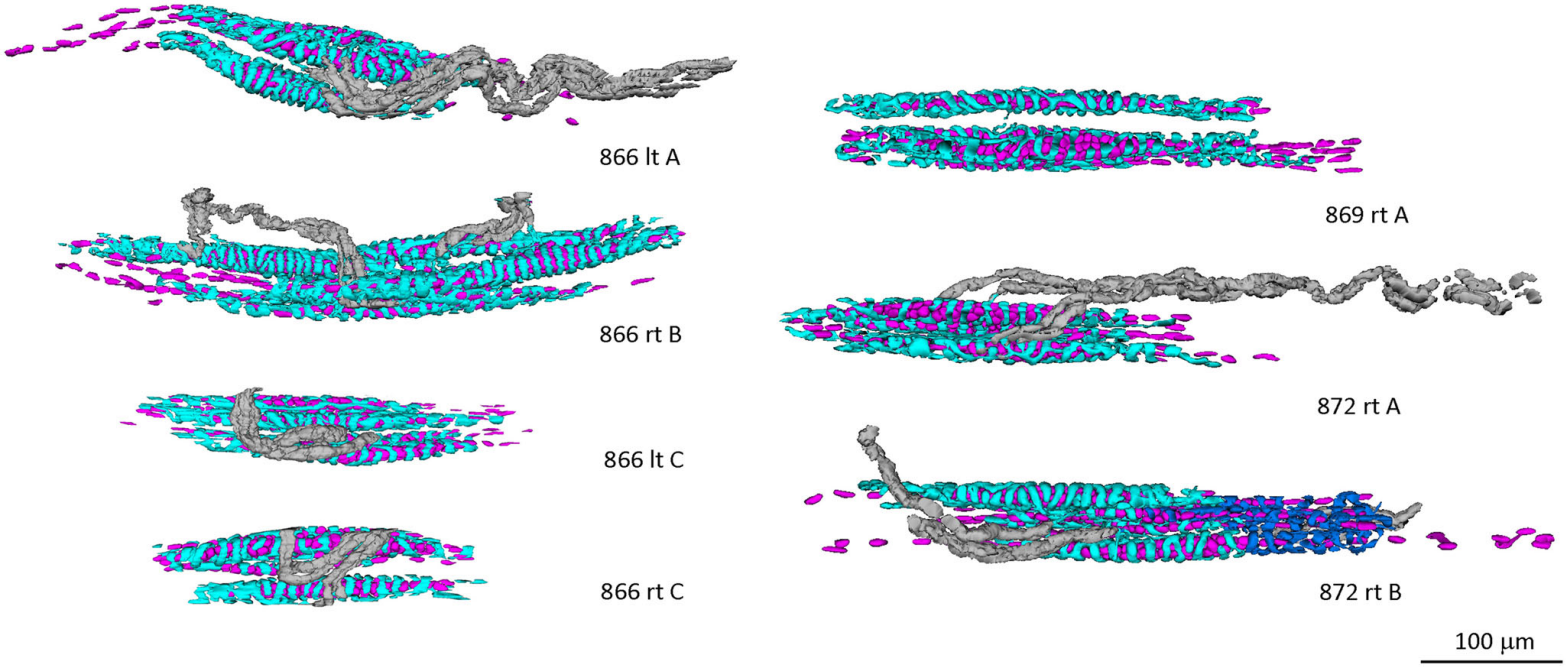
Virtual reconstructions of the sensory terminals and associated intrafusal fibre nuclei of the equatorial regions of seven spindles from the left (lt) or right (rt) tenuissimus muscles from three adult cats. The spindles were located in the proximal (A), middle (B) or distal (C) thirds of the muscles. Colours are as follows: light blue, primary‐ending terminals; dark blue, terminals of an incomplete secondary ending in 872 rt B; purple, equatorial nuclei; grey, myelinated pre‐terminal branches.

Seven spindles were sufficiently complete to allow the tracing and reconstruction of the sensory terminals of their primary endings and of the underlying equatorial nuclei (Figure 3; Supporting Information Video). Intrafusal fibres were classified firstly into bag and chain fibres and secondly into bag_1_ and bag_2_ fibres using several criteria, including the arrangement of the nuclei in bags or chains, and the relative spacing and prominence of their sensory terminals (Banks et al., 1982). According to these criteria, each spindle contained one bag_1_ fibre and four to six chain fibres; five spindles contained one bag_2_ fibre, and the remaining two spindles contained a second bag_2_ fibre. It is notable that the three largest spindles by intrafusal fibre complement (b_1_b_2_6c, b_1_2b_2_6c and b_1_2b_2_6c) were all from the same animal. Virtual reconstructions were made using Boissonnat surface rendering in RECONSTRUCT. For each traced profile, the length of the tracing and the area contained by it provided the raw quantitative data. Volumes were obtained, using the Cavalieri principle, by integrating the profile areas, taking the nominal section thickness of 1 μm.

**FIGURE 4:**
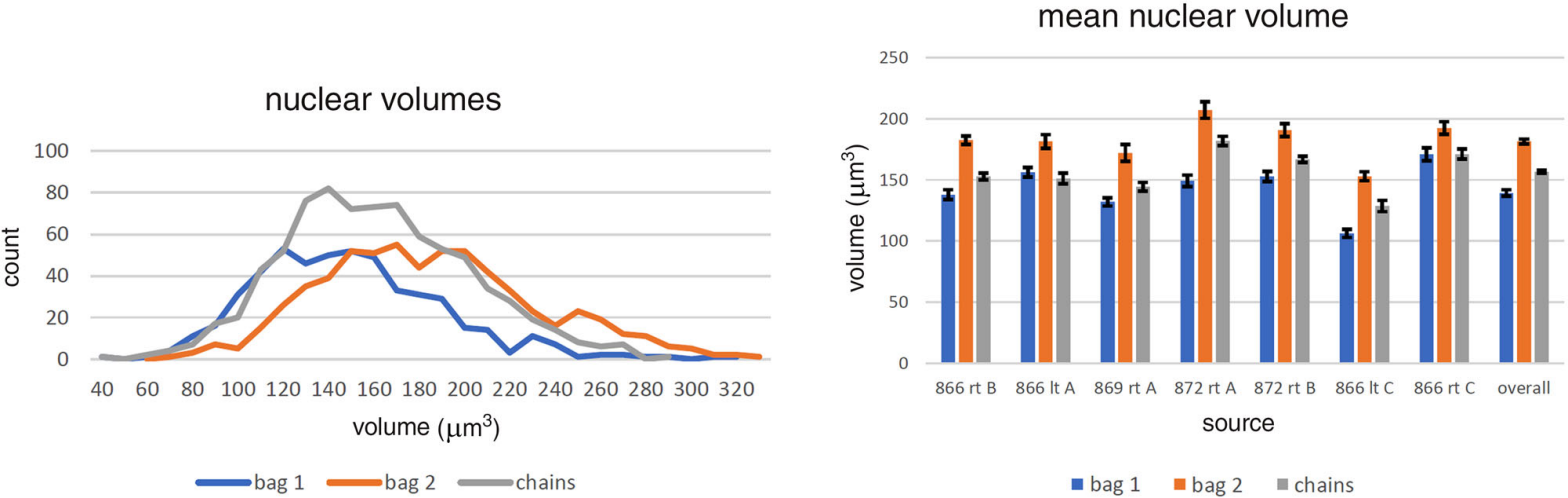
The left panel shows the distribution of equatorial nuclear volumes from all seven spindles in the reconstructions of Figure 3. The right panel shows the nuclear volumes (mean ± SEM) of the equatorial nuclei of each type of intrafusal fibre, from each spindle. Bag_2_ fibres consistently exhibit equatorial nuclei with the greatest mean volume, followed, in most cases, by the chain fibres.

Total numbers of nuclei associated with each primary ending ranged from 250 to 485; further details are set out in Table 1. Overall mean nuclear volumes (in micrometres cubed), together with the range for the means for individual spindles, were as follows: bag_1_, 139.3 (106.2–170.9); bag_2_, 181.4 (153.0–207.1); and chain, 156.7 (128.6–181.8) (Figure 4). By comparison, 14 subsarcolemmal nuclei in juxta‐equatorial regions of the bag_1_ or bag_2_ fibres from five of the spindles had a mean volume of 109.2 μm^3^, and 40 Schwann cell nuclei of preterminal myelinated segments of Ia afferents from six of the spindles had a mean volume of 137.7 μm^3^.

**FIGURE 5:**
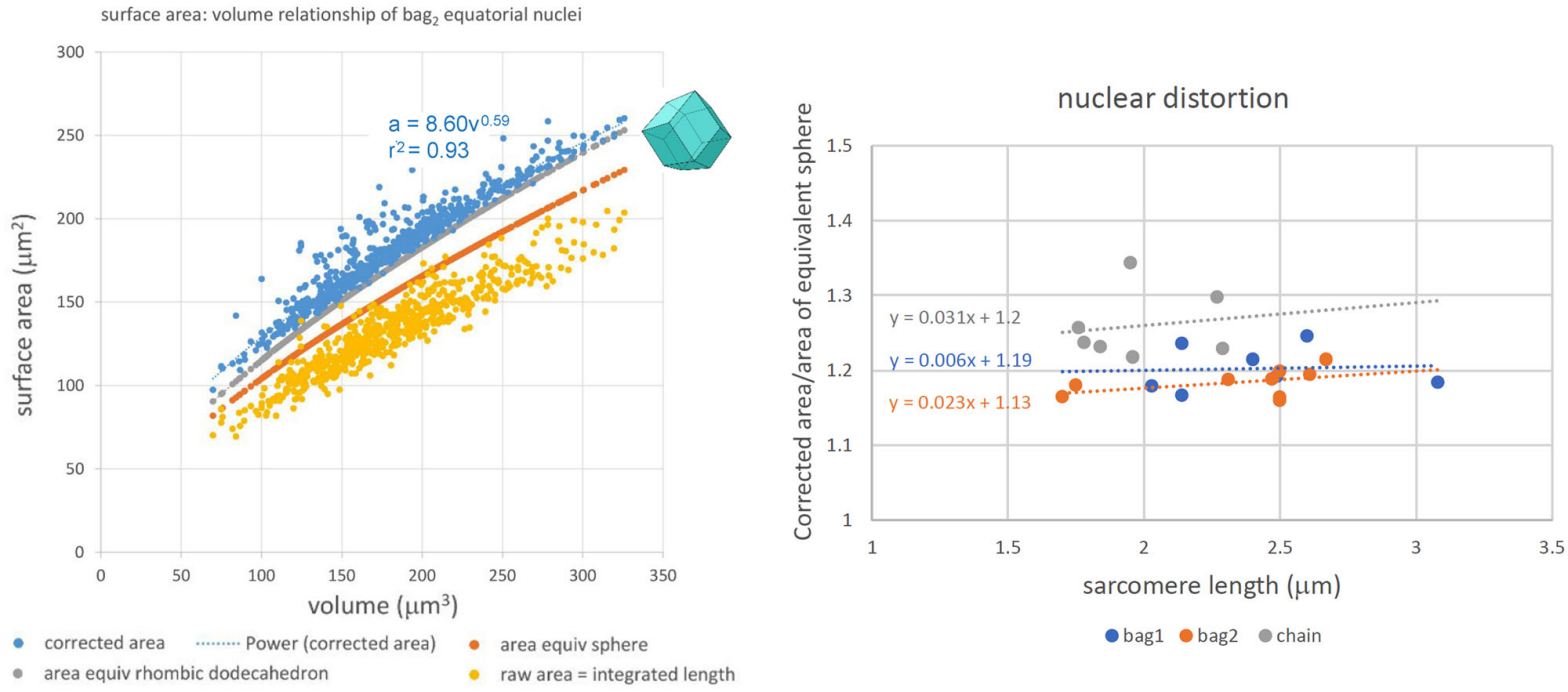
The left panel shows the surface area of each equatorial nucleus of all nine bag_2_ fibres, plotted against its volume. The distribution of the complete raw data (yellow) in relationship to the surface areas of spheres having the same volumes (orange) demonstrates the necessity for a correction as described in the main text. Corrected values (blue, with fractional power regression) approach the limit of the surface areas of rhombic dodecahedra having the same volumes and which can be close‐packed (grey). Similar results were obtained for bag_1_ and chain fibres. The right panel shows plots of equatorial nuclear distortion, as measured by the ratio of corrected surface area to the area of the equivalent sphere, against mean juxta‐equatorial sarcomere length, for the three types of intrafusal fibres.

Whether in a chain or in a bag with myotubes, the equatorial nuclei are mostly packed closely together, with interfaces that are flat (see below). Each nucleus must therefore have a larger surface area than a sphere with the same volume, whilst remaining, at least in most cases, three‐dimensionally convex. Integration of the lengths of the profile traces provides, by analogy to the Cavalieri principle for volumes, a first approximation of the surface area of the nucleus. Plotting the raw measure of surface area against the Cavalieri volume, together with the surface area of the equivalent sphere, clearly demonstrated the necessity to apply a correction to take account of the effect of section thickness, because the raw measure of surface area was, in almost all cases, less than the surface area of the equivalent sphere. The method of correction, implemented in EXCEL, was to replace the measured profile perimeter, which represented the surface area of the edge of a disc of 1 μm thickness and parallel faces each with the area of the profile, with the surface area of the edge of a pyramidal frustrum with parallel faces of successive profile areas, beginning and ending with profile areas equal to zero. Plotting the corrected value for each nucleus results in a new minimal value that approaches that of the simplest semiregular solid of the same volume that can be closely packed, the rhombic dodecahedron (Figure 5). The ratio of the corrected area to that of the equivalent sphere can be taken as a measure of the amount of nuclear distortion (or deviation from sphericity) attributable to the close packing of the nuclei. Overall mean ratios, together with the range for the means for individual spindles, were as follows: bag_1_, 1.20 (1.17–1.25); bag_2_, 1.18 (1.16–1.21); and chain, 1.26 (1.22–1.34). There was no evidence of a significant change in nuclear distortion in relationship to mean juxta‐equatorial sarcomere length (Figure 5).

**FIGURE 6:**
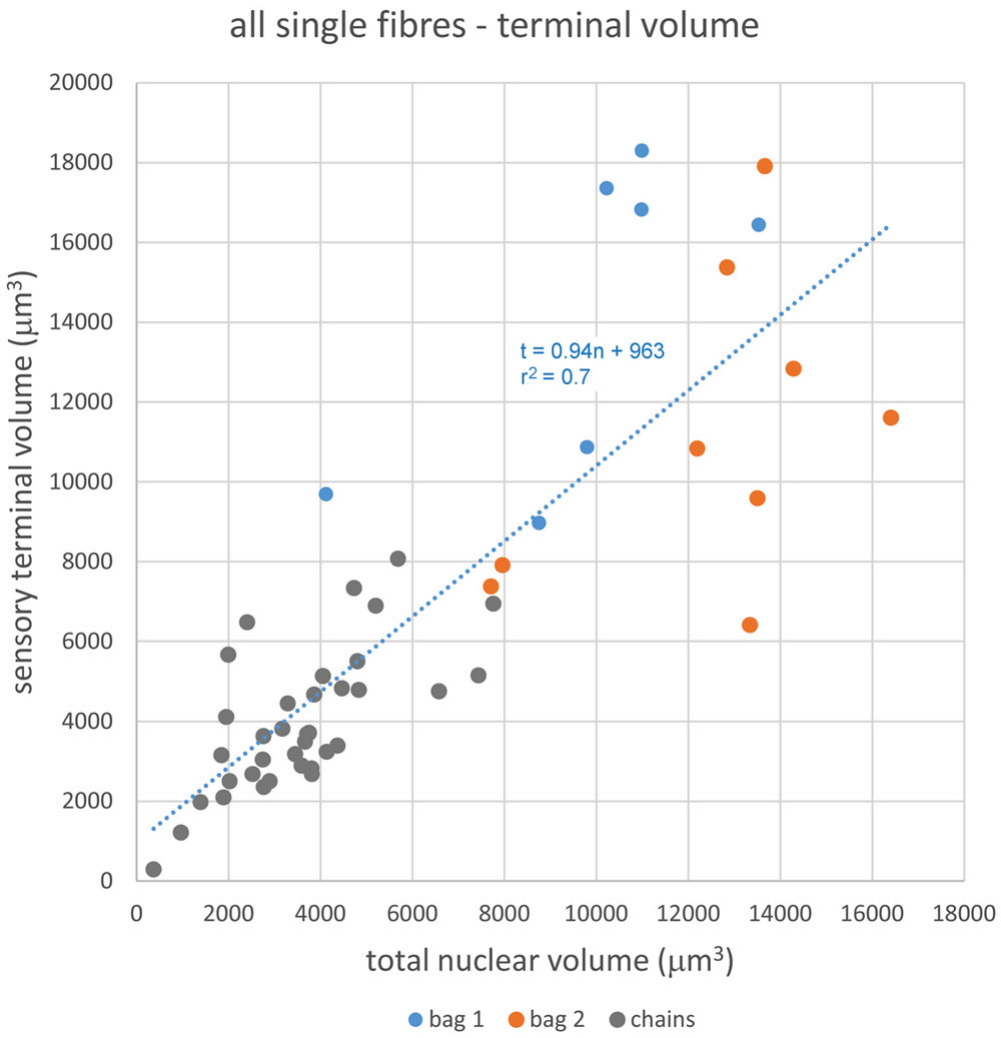
The relationship between primary sensory terminal volume and total equatorial nuclear volume for all individual intrafusal fibres from the seven reconstructed spindles of Figure 3.

The well‐known association between primary sensory terminals and equatorial nuclei is evident in the reconstructions, but the measured volumes provide a remarkable, but perhaps surprising, quantitative relationship. Taking the intrafusal fibre population as a whole, terminal and nuclear volumes for each fibre are closely similar, with a linear regression of 0.94, although the bag fibres tend to deviate in a slightly positive (bag_1_) or negative (bag_2_) direction from the overall regression (Figure 6).

**FIGURE 7:**
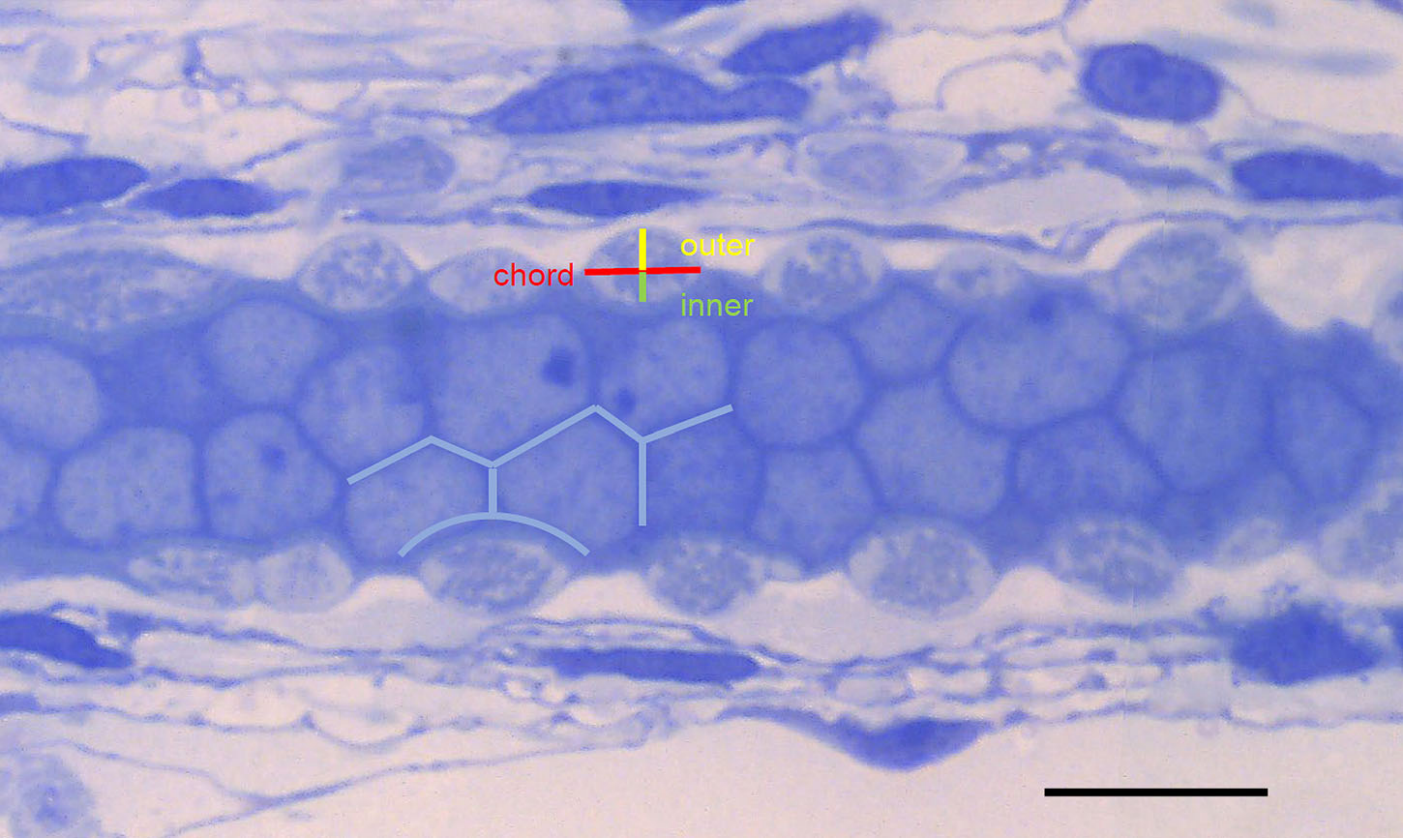
A representative section of a bag_1_ fibre (872 rt B, section 63) used to reconstruct the primary endings of Figure 3, showing the three measured parameters of lentiform terminal profiles, chord length (red), and outer (yellow) and inner (green) segment heights. Note also the generally flat interfaces between equatorial nuclei and their adoption of slightly concave faces when distorted by closely overlying terminals (blue lines). Length of scale bar (10 μm).

**FIGURE 8:**
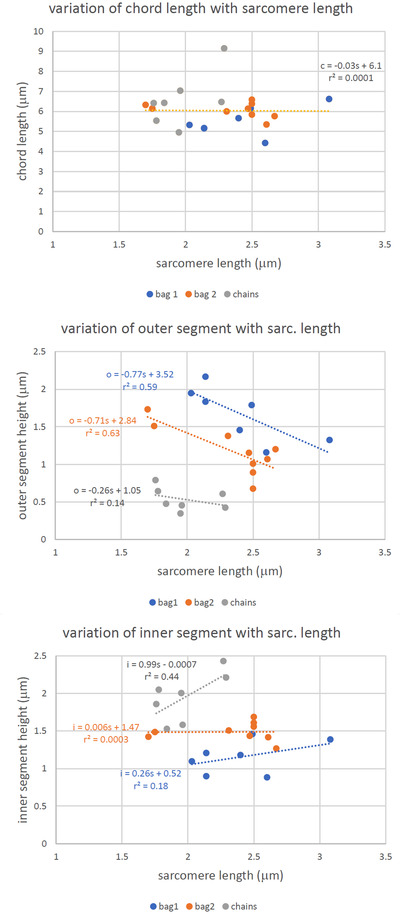
Plots showing the relationships between chord length (top), outer segment height (middle) and inner segment height (bottom) against mean juxta‐equatorial sarcomere length for each of the three types of intrafusal fibres.

Terminal profiles in longitudinal sections passing close to or through the diameter of each intrafusal muscle fibre exhibited lenticular shapes, consisting of an outer segment, covered externally by basal lamina continuous with that of the muscle fibre, and an inner segment in close contact with the unit membrane or plasmalemma of the muscle fibre (as first described by Banks, 1986). The segments approximate closely to circular arcs sharing a common chord in the line of the adjacent sarcolemma (basal lamina + plasmalemma). The shapes of 10 such profiles from each type of intrafusal fibre from all of the spindles were quantified by measuring the chord length and the heights of the outer and inner arcs (Figure 7). Linear regression of mean chord length (*c*) against mean juxta‐equatorial sarcomere length (*s*) was essentially flat for all three types of fibre taken together, as follows: *c* = −0.03*s* + 6.11 (*r* ^2^ = 0.0001) (Figure 8). The heights of the outer (*o*) and inner (*i*) arcs, however, dependent on both intrafusal fibre type and mean juxta‐equatorial sarcomere length. Mean values of *o* were consistently greatest for bag_1_ fibres, intermediate for bag_2_ fibres and least for chain fibres. All three cases showed a reduction in mean *o* with mean sarcomere length, as follows: bag_1_ *o* = −0.77*s* + 3.52 (*r* ^2^ = 0.59); bag_2_ *o* = −0.71*s* + 2.84 (*r* ^2^ = 0.63); and chain *o* = −0.26*s* + 1.25 (*r* ^2^ = 0.14) (Figure 8). Mean values of *i* were consistently least for bag_1_ fibres, intermediate for bag_2_ fibres and greatest for chain fibres. For bag_1_ and chain fibres, mean *i* increased with mean sarcomere length, whereas the relationship was essentially flat for bag_2_ fibres, as follows: bag_1_ *i* = 0.26*s* + 0.52 (*r* ^2^ = 0.18); bag_2_ *i* = 0.006*s* + 1.47 (*r* ^2^ = 0.0003); and chain *i* = 0.99*s* + 0.0007 (*r* ^2^ = 0.44) (Figure 8).

### 2.4 Discussion

As early as 1897, Cipollone (1897) gave a remarkably full description of the equatorial nuclei of intrafusal muscle fibres and, notwithstanding their highly unusual appearance, almost to the point of not being recognizable as nuclei, concluded that they ‘devono interpretarsi come nuclei muscolari modificati’ (must be interpreted as modified muscle fibre nuclei). It is a curious fact, but despite long familiarity with these nuclei, the functional significance of their unique, closely packed grouping has rarely been commented on. We may now see that their modification includes being both highly euchromatic and considerably larger than the transcriptionally active subsarcolemmal nuclei of the same fibres. Euchromasia is normally associated with extensive transcription, but given that most of these nuclei lack any obvious translation apparatus nearby, it seems highly unlikely that expression of a large proportion of their genome is their main function. They are, moreover, also distinct in their gene expression from the remaining nuclei of the intrafusal fibres (Kim et al., 2020). Of the nine genes identified as characterizing the equatorial nuclei with above average expression (*Calcrl*, *Etv4*, *Erf*, *Pdgfa*, *Fbxw7*, *Frmd4b*, *Cpne7*, *Ctnnbip1* and *Daam2*), some code for transcription factors, and others appear as tumour suppressors in the databases, but none has any obvious role to play in equatorial nuclear function. Could it be that the main function of these nuclei is mechanical; simply to be large, to remain packed closely together under the sensory terminals, be deformable, resistant to compression and thus to provide, developmentally, a readily available, compliant, elastic substrate for sensory terminal function? That they are, indeed, compliant is attested by their adoption of any shape determined by neighbouring structures (in most cases, of course, other nuclei).

The sensory terminals, too, are compliant, as indicated by their changes in shape, with *o* decreasing and *i* increasing according to sarcomere length, irrespective of fibre type. These changes are entirely consistent with a model in which the terminals are deformed by differential tension in the basal lamina and underlying muscle fibre (Banks, 1986, 2015), with basal lamina tension increasing more rapidly than muscle fibre tension. The differences in shape according to fibre type are then attributable to the different mechanical properties of the fibres. How the tension is transmitted to the basal lamina remains uncertain. It might be attributable, in part, to elongation of the muscle fibres, but there appears to be a standing tension, presumably derived from connective tissue, which maintains the sensory region straight even when the spindle is shortened to such an extent that the intrafusal fibres are thrown into folds in the adjacent regions (e.g., evident in 866 lt A). By assuming that the sensory terminals are constant‐volume structures and that the effect of their annulospiral shape is to minimize length changes (Banks, 2015), attention is focused on the changes in profile shape as imparting the terminal membrane stretch necessary for mechanosensory transduction.

**FIGURE 9:**
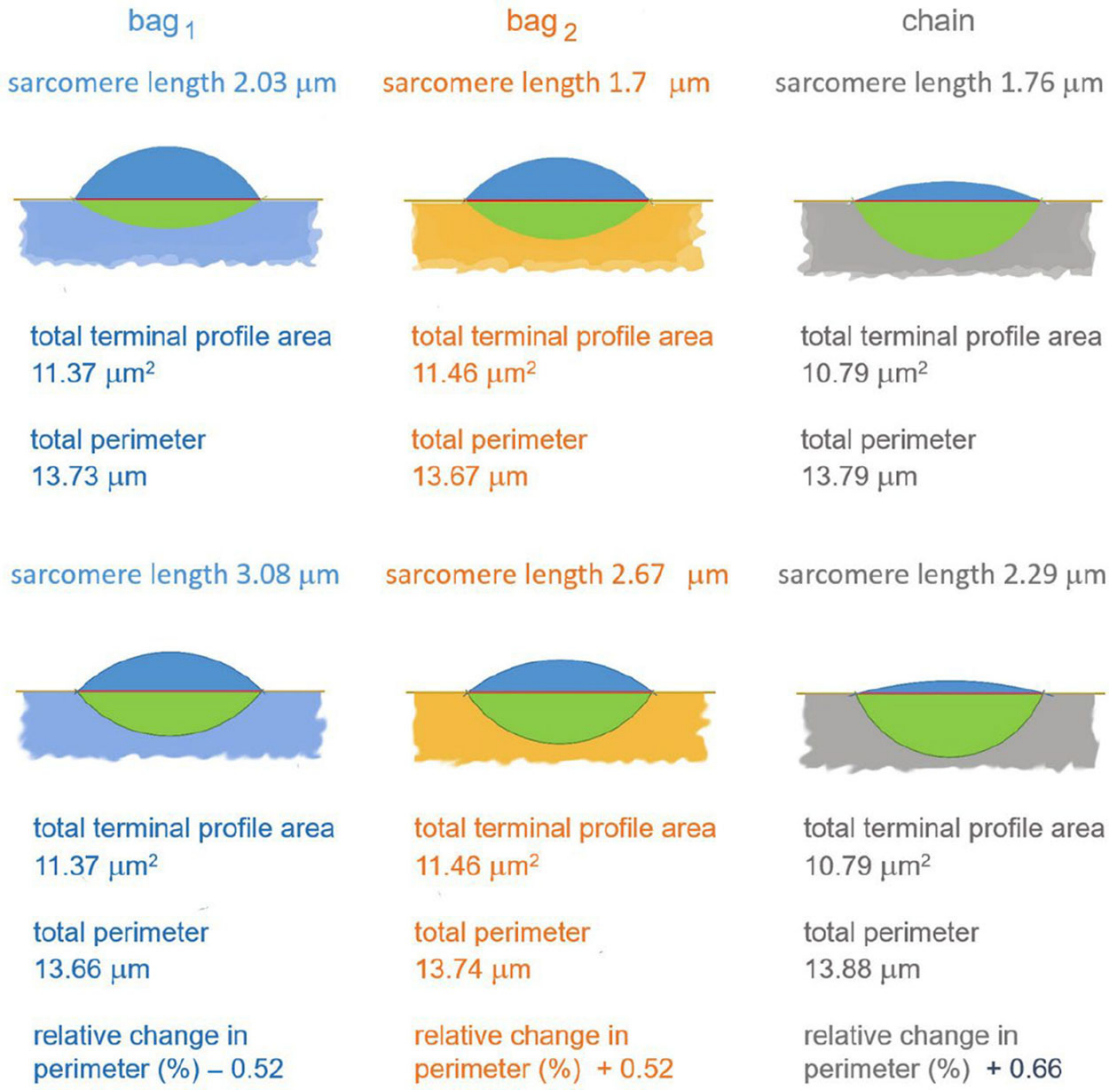
Schematic diagrams to show the estimated changes in shape of the lentiform profiles of primary sensory terminals, based on the regression relationships of Figure 8. Full details are given in the main text.

The model requires that, as the terminal profiles change shape, they maintain a constant area, and the perimeter is then a proxy for terminal membrane stretch. The lenticular profiles approximate closely to two circular segments, whose areas can be calculated from *c*, *o* and *i*. Given that the raw data were all obtained from individual spindles, often from different animals, unbiased estimates of the segment areas, hence total profile areas, were first calculated using values of *c*, *o* and *i* derived from the regression relationships and the minimum and maximum mean sarcomere length for each fibre type. Adjusting these minimum and maximum areas to the mean for each pair allowed corresponding adjustments to be back‐calculated for *c*, *o* and *i* (in effect, slightly altering the regression slope in each case), from which the perimeters of the now equal area profiles could be calculated. In the case of the bag_2_, for example, the total terminal profile area was estimated as 11.46 μm^2^, and at the minimum observed mean sarcomere length of 1.7 μm the total perimeter was 13.67 μm. At the maximum observed mean sarcomere length of 2.67 μm, the total perimeter was 13.74 μm, an increase of 0.52%. These and similar details for bag_1_ and chain fibres are represented diagrammatically in Figure 9.

Comparison with my earlier results (Banks, 1986) should not be pressed too far, at least in part because that sample consisted of only three spindles, all necessarily from different animals. But there is a clear discrepancy in the case of the radius of curvature of the inner circular arc (*r_i_*) of the bag_1_ fibres. The present result, based on the regression relationship as described above, has values of *r_i_* of 5.4 μm at the minimum mean sarcomere length of 2.03 μm, and *r_i_* of 3.8 μm at the maximum length of 3.08 μm, whereas the earlier results were 5.2 μm at 2.2 μm, 20.1 μm at 2.5 μm and 31.9 μm at 2.6 μm. In the differential tension model described above, these last two very high values would signify a relatively large tension in the bag_1_ fibre. That this might, indeed, have been the case can probably be attributed to the different preparative techniques used. In the present study, the muscles were entire, in situ and completely passive at the point of fixation, whereas in the earlier study the three spindles were all part of the histophysiological work of Banks et al. (1978). In each case, the spindles had been exposed, in part, with intact nerve and blood supplies, and one or two dynamic γ axons had been stimulated repetitively at ≤110 impulses/s, thereby generating active tension in the bag_1_ fibres.

## 3 CONCLUDING REMARKS

In the first part of this paper, I have given a brief account of a few of the advances made during the 50 years I have spent wandering through unknown spindle‐land, and to which I was able to make some contributions. However important the particular advances themselves are, I now wish to conclude by emphasizing the general necessity of maintaining a sceptical mind and to beware of taking apparently established matters or familiar observations for granted. An apparently established matter that I took for granted for too long was the general acceptance that (1) different muscles have different abundances of spindles, and (2) so‐called spindle density was a good measure of spindle abundance. After a decade or so of wandering, while continuing to recognize that spindles are more abundant in some muscles than others, I became sceptical of spindle density as a valid measure of abundance, eventually presenting an alternative approach based on the principle of allometry during my fourth decade (Banks, 2006). The presence of the equatorial nuclei of intrafusal muscle fibres and their close association with the primary sensory ending are very familiar observations that I certainly took for granted until recently. But such clusters of large nuclei are extremely unusual and perhaps unique in mammalian cell structure, so what is their function?

In the second part of this paper, I have presented a new quantitative analysis of the morphology of the nuclei and of the primary sensory terminals to draw attention to the most unusual nature of the nuclei and to raise the possibility that they might be specialized primarily for a mechanical function.

## AUTHOR CONTRIBUTIONS

Sole author.

## CONFLICT OF INTEREST

None declared.

## FUNDING INFORMATION

None.

## Supporting information

Supplementary movie 1
