# Supplementary figures and images for: There and back again: 50 years of wandering through terra incognita fusorum
Source: Exp Physiol. 2023 Jan 11;109(1):6–16. doi: 10.1113/EP090760 (PMC10988739; doi:10.1113/EP090760)

## Slide 1
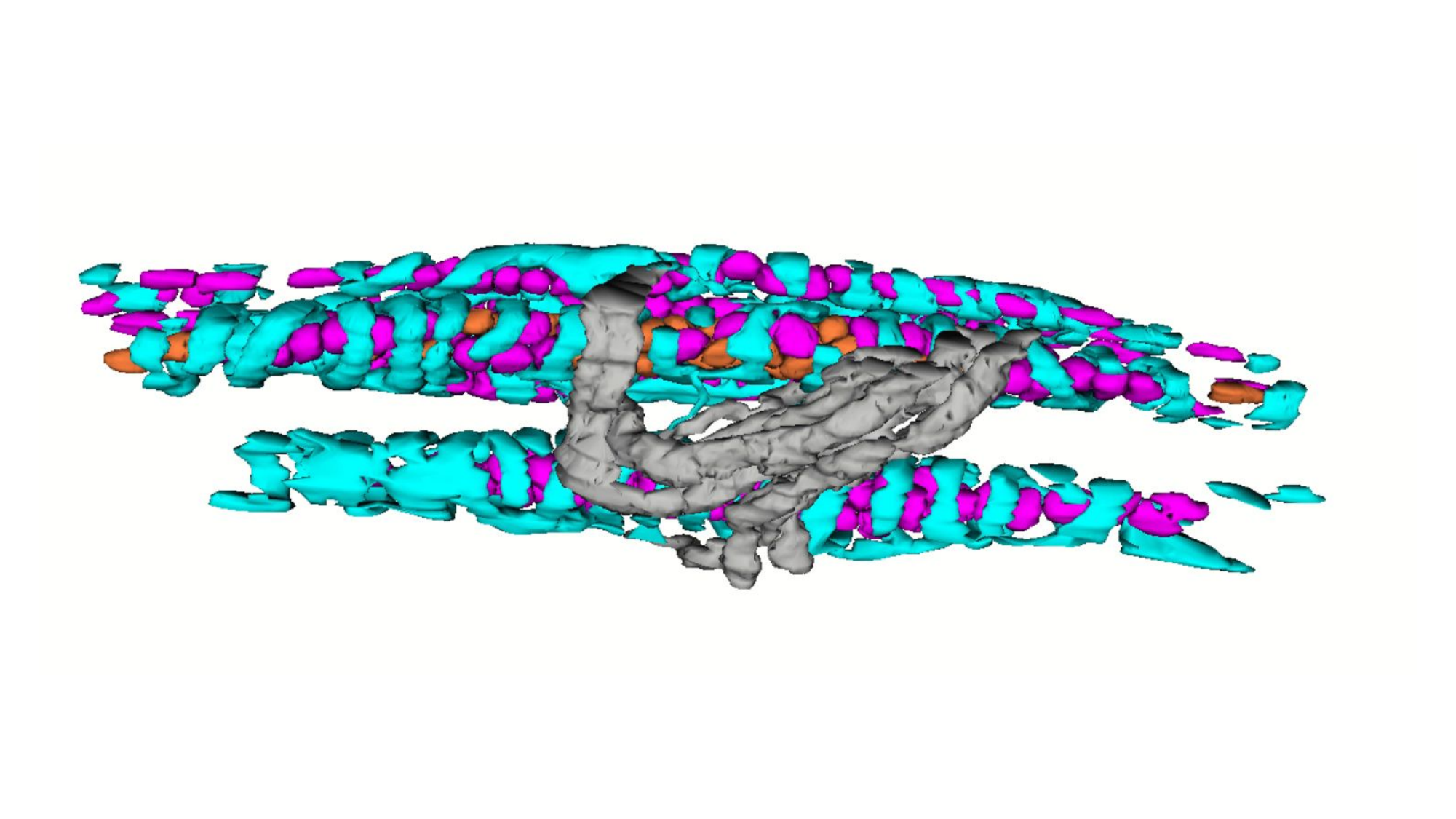

Supplement: Supplementary file 1 — Supplementary movie 1 [file EPH-109-6-s001.pptx]
